# European Birth Cohorts for Environmental Health Research

**DOI:** 10.1289/ehp.1103823

**Published:** 2011-08-29

**Authors:** Martine Vrijheid, Maribel Casas, Anna Bergström, Amanda Carmichael, Sylvaine Cordier, Merete Eggesbø, Esben Eller, Maria P. Fantini, Mariana F. Fernández, Ana Fernández-Somoano, Ulrike Gehring, Regina Grazuleviciene, Cynthia Hohmann, Anne M. Karvonen, Thomas Keil, Manolis Kogevinas, Gudrun Koppen, Ursula Krämer, Claudia E. Kuehni, Per Magnus, Renata Majewska, Anne-Marie Nybo Andersen, Evridiki Patelarou, Maria Skaalum Petersen, Frank H. Pierik, Kinga Polanska, Daniela Porta, Lorenzo Richiardi, Ana Cristina Santos, Rémy Slama, Radim J. Sram, Carel Thijs, Christina Tischer, Gunnar Toft, Tomáš Trnovec, Stephanie Vandentorren, Tanja G.M. Vrijkotte, Michael Wilhelm, John Wright, Mark Nieuwenhuijsen

**Affiliations:** 1 Centre for Research in Environmental Epidemiology (CREAL), Barcelona, Spain; 2 Hospital del Mar Research Institute (IMIM), Barcelona, Spain; 3 Spanish Consortium for Research on Epidemiology and Public Health (CIBERESP), Spain; 4 Institute of Environmental Medicine, Karolinska Institutet, Stockholm, Sweden; 5 School of Social and Community Medicine, University of Bristol, United Kingdom; 6 French National Institute of Health and Medical Research (INSERM), U625; University of Rennes I; IFR140; Rennes, France; 7 Division of Epidemiology, Norwegian Institute of Public Health, Oslo, Norway; 8 Department of Dermatology and Allergy Centre, Odense University Hospital, Denmark; 9 Department of Public Health, University of Bologna, Bologna, Italy; 10 Laboratory of Medical Investigations, San Cecilio University Hospital, University of Granada, Granada, Spain; 11 University of Oviedo, Asturias, Spain; 12 Institute for Risk Assessment Sciences, Utrecht University, Utrecht, the Netherlands; 13 Department of Environmental Sciences, Vytautas Magnus University, Kaunas, Lithuania; 14 Institute of Social Medicine, Epidemiology and Health Economics, Charité University Medical Center, Berlin, Germany; 15 Department of Environmental Health, National Institute for Health and Welfare, Kuopio, Finland; 16 National School of Public Health, Athens, Greece; 17 Environmental Risk and Health Unit, Flemish Institute of Technological Research (VITO), Mol, Belgium; 18 IUF - Leibniz Research Institute for Environmental Medicine (IUF), Düsseldorf, Germany; 19 Institute of Social and Preventive Medicine (ISPM), University of Bern, Bern, Switzerland; 20 Department of Infection, Immunity and Inflammation, University of Leicester, Leicester, United Kingdom; 21 Epidemiology and Preventive Medicine, Jagiellonian University Medical College, Kraków, Poland; 22 Section for Social Medicine, Department of Public Health, University of Copenhagen, Copenhagen, Denmark; 23 Department of Social Medicine, Medical School, University of Crete, Heraklion, Greece; 24 Department of Occupational Medicine and Public Health, The Faroese Hospital System, Tórshavn, Faroe Islands; 25 Department of Urban Environment, Netherlands Organisation for Applied Scientific Research (TNO), Utrecht, the Netherlands; 26 The Generation R Study Group, Erasmus Medical Center Rotterdam, Rotterdam, the Netherlands; 27 Nofer Institute of Occupational Medicine, Department of Environmental Epidemiology, Lodz, Poland; 28 Department of Epidemiology Regional Health Service, Lazio, Rome, Italy; 29 Cancer Epidemiology Unit, CPO-Piemonte and University of Turin, Turin, Italy; 30 Department of Hygiene and Epidemiology, University of Porto Medical School and Institute of Public Health, University of Porto, Porto, Portugal; 31 INSERM and Grenoble University, Team of Environmental Epidemiology Applied to Reproduction and Respiratory Health, Institut Albert Bonniot (U823), Grenoble, France; 32 Institute of Experimental Medicine AS CR, Prague, Czech Republic; 33 Department of Epidemiology, School for Public Health and Primary Care (CAPHRI), Maastricht University, Maastricht, the Netherlands; 34 Helmholtz Zentrum, München, German Research Centre for Environmental Health, Institute of Epidemiology, Neuherberg, Germany; 35 Department of Occupational Medicine, Aarhus University Hospital, Aarhus, Denmark; 36 Slovak Medical University, Bratislava, Slovakia; 37 National Institute of Public Health Surveillance (InVS), Saint Maurice, France; 38 Department of Public Health, Academic Medical Centre, Amsterdam, The Netherlands; 39 Hygiene, Social and Environmental Medicine, Ruhr-University, Bochum, Germany; 40 Bradford Institute for Health Research, Bradford, United Kingdom

**Keywords:** birth cohorts, child health, environmental exposures, Europe, review

## Abstract

Background: Many pregnancy and birth cohort studies investigate the health effects of early-life environmental contaminant exposure. An overview of existing studies and their data is needed to improve collaboration, harmonization, and future project planning.

Objectives: Our goal was to create a comprehensive overview of European birth cohorts with environmental exposure data.

Methods: Birth cohort studies were included if they *a*) collected data on at least one environmental exposure, *b*) started enrollment during pregnancy or at birth, *c*) included at least one follow-up point after birth, *d*) included at least 200 mother–child pairs, and *e*) were based in a European country. A questionnaire collected information on basic protocol details and exposure and health outcome assessments, including specific contaminants, methods and samples, timing, and number of subjects. A full inventory can be searched on www.birthcohortsenrieco.net.

Results: Questionnaires were completed by 37 cohort studies of > 350,000 mother–child pairs in 19 European countries. Only three cohorts did not participate. All cohorts collected biological specimens of children or parents. Many cohorts collected information on passive smoking (*n* = 36), maternal occupation (*n* = 33), outdoor air pollution (*n* = 27), and allergens/biological organisms (*n* = 27). Fewer cohorts (*n* = 12–19) collected information on water contamination, ionizing or nonionizing radiation exposures, noise, metals, persistent organic pollutants, or other pollutants. All cohorts have information on birth outcomes; nearly all on asthma, allergies, childhood growth and obesity; and 26 collected information on child neurodevelopment.

Conclusion: Combining forces in this field will yield more efficient and conclusive studies and ultimately improve causal inference. This impressive resource of existing birth cohort data could form the basis for longer-term and worldwide coordination of research on environment and child health.

It is well recognized that the fetus and infant are especially vulnerable to the effects of environmental risk factors that disrupt developmental processes, due to *a*) critical windows of vulnerability that occur during the rapid growth and development of organs and systems, *b*) immaturities in metabolism, and *c*) greater intake and absorption of noxious agents in children relative to their body weight (Grandjean et al. 2008). Chemical, physical, and biological hazards in the environment may lead to serious health problems at birth and during childhood, ranging from premature birth, low birth weight, and congenital anomalies to respiratory diseases, childhood cancer, learning disabilities, behavioral problems, and possibly even obesity (Trasande et al. 2009; Wigle et al. 2007). The economic and societal costs associated with nonoptimal child health and development are substantial. Furthermore, the effects of exposure to environmental risk factors may manifest themselves throughout a lifetime and even over generations. Common physical and mental diseases in adult life appear to have part of their origin in early life (Gillman et al. 2007; Kuh et al. 2003).

Epidemiological studies worldwide have investigated the role of early environmental contaminant exposures on pregnancy outcomes and child health (Wigle et al. 2007). Many have suffered from biases due to retrospective study designs, surrogate exposure and outcome measures, and incomplete confounder data. Pregnancy and birth cohort studies are ideally suited to improve causal inference in this field, because they are designed to study the impacts of early exposures prospectively and at multiple time points during development of the child. Furthermore, birth cohort studies usually collect biological material from mothers and children, enabling the measurement of biomarkers of exposure, early effect, or susceptibility. In the last two decades, new pregnancy and birth cohorts have been set up across Europe (e.g., Golding et al. 2001; Hofman et al. 2004; Magnus et al. 2006; Olsen et al. 2001; Raynor 2008; Richiardi et al. 2007), in North America, and elsewhere (Brown et al. 2007; Herbstman et al. 2010; Marks et al. 2010; Richter et al. 2011), and more are being planned (Hirschfeld et al. 2011). Many cohort studies include assessments of environmental pollutant exposure, but not all have large expertise in this area. Although it is clear that individual cohorts are able to make, and have made, important contributions to understanding environmental causes of childhood disease and ill health, it is becoming increasingly clear that their full potential can be realized only with collaboration across large regions (Kogevinas et al. 2004).

The European Union (EU) has funded the Environmental Health Risks in European Birth Cohorts (ENRIECO) project to coordinate birth cohort research in Europe in the area of environmental contaminant exposures (ENRIECO 2009). ENRIECO recognized that a comprehensive overview of birth cohorts and their data was needed as a first step toward improved collaboration, future project planning, and effective use of existing data. Currently, no inventory of environmental exposure data exists. The aim of this review is to provide an overview of European birth cohorts with data on environmental contaminant exposures and health outcomes.

## Methods

*Definition and identification of cohorts*. Birth cohort studies were included in the project if they *a*) collected data on at least one environmental contaminant exposure topic (air pollution, water contamination, pesticides, metals, persistent organic pollutants, other chemical pollutants, noise, radiations, and allergens and biological organisms); *b*) started enrollment of mothers into the cohort during pregnancy or at birth; *c*) included in their protocol at least one follow-up point after birth with direct contact with mothers and children; *d*) included at least 200 mother–child pairs (a low sample size limit was chosen so small studies with relevant exposure data or based in less well-covered regions were not excluded); and *e*) were based in a European country. Collection of data on an environmental exposure topic included data collected through questionnaires, environmental sampling, spatial maps and models, personal monitoring, biomarker measurements, or a combination of these methods. The mere availability of residential location (addresses and geocodes) was not sufficient for inclusion. All cohorts fitting the criteria were considered regardless of when they started, when they conducted their most recent follow-up, whether data had been published, or whether recruitment of subjects was ongoing.

Cohorts were identified through a variety of sources: www.birthcohorts.net (Birthcohorts.net 2010), a searchable Web site that contains basic protocol information for a number of birth cohorts; European projects using birth cohort data; and personal contacts. Publications and Web sites of identified cohorts were checked to see whether the cohort appeared to match the criteria of the inventory. All cohorts identified as potentially eligible between 1 March 2009 (start of the project) and 31 December 2010 were approached.

*Inventory questionnaire.* An inventory questionnaire was designed to collect detailed information from the cohorts on biological sample collection, assessment of environmental exposures, assessment of child health outcomes of interest, and basic information on collection of other variables. The full questionnaire can be found in Supplemental Material, Annex 1 (http://dx.doi.org/10.1289/ehp.1103823). The questionnaire was divided into four sections:

Basic protocol description: protocol description, basic data, and biological sample collection scheme.
Exposure assessment: air pollution (outdoor and indoor), water contamination, allergens and biological organisms, metals, pesticides, radiations (ionizing, ultraviolet, and nonionizing), tobacco smoke (active and passive), noise, persistent organic pollutants, occupation (maternal and paternal), and other chemical compounds. Each section asked for the exposure assessment methods used, numbers of subjects assessed, and time point of assessment.
Health outcome assessment: reproduction and birth outcomes, neurodevelopment, allergies and asthma, cancer, child growth, and metabolic syndrome indicators such as blood pressure, cholesterol, triglycerides, insulin, or glucose. Each section asked which outcomes were assessed, by which methods (if relevant), how many subjects were assessed, and the time point of assessment.
Other information: mainly yes/no questions on collection of genotype data, residential history, time–activity patterns, sociodemographic variables, breast-feeding, diet, physical exercise, medical history, and anthropometry.

The questionnaire was sent to principal investigators of each identified cohort, who were asked to distribute specific sections to the appropriate researchers and data managers within their cohort. The completed questionnaire was checked and, if necessary, the cohort study teams were contacted again to correct errors and complete information. Cohorts that did not reply to the first e-mail were reminded repeatedly by e-mail or phone. Cohorts were invited to two workshops during the project, in which the aims and objectives were presented.

A Microsoft Access database was constructed to contain the responses from the cohorts. All cohorts were asked to check this database for errors before making it public. The last corrections and updates were made in February 2011. A Web-based searchable inventory is now publicly available on www.birthcohortsenrieco.net (ENRIECO 2011) and also accessible through www.birthcohorts.net (Birthcohorts.net 2010).

## Results

**Figure 1:**
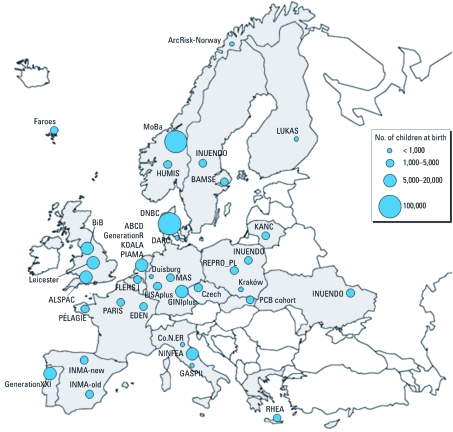
European birth cohort studies collaborating in ENRIECO.

A total of 46 birth cohort studies were identified as potentially eligible based on publications, Web sites, and word of mouth. Six of these were excluded after completion of questionnaires, because they did not match the eligibility criteria: Three did not assess environmental exposures, and three did not have a follow-up point after birth. Another three birth cohorts did not return the questionnaires (Table 1). The 37 included cohorts, totaling > 350,000 study subjects, are situated in 19 European countries, principally in Northern and Western Europe (Figure 1). Five of the cohorts are in Eastern Europe and seven in Southern Europe (based on the European regions classified in the United Nations geoscheme). Twenty-five of the 37 cohorts defined themselves as regionally based studies, six as nationally based, four as hospital based, and two as studying selected populations [farmers in the LUKAS cohort and high-risk allergic children in the Multicentre Allergy Study (MAS)]. Two studies, the Danish National Birth Cohort (DNBC) and the Norwegian Mother and Child Study (MoBa) have recruited around 100,000 mother–child pairs each (Table 1). Ten cohorts have recruited between 5,000 and 20,000 pairs, 18 between 1,000 and 5,000 pairs, and seven cohorts concern < 1,000 pairs.

**Table 1:** General description of European birth cohorts with environmental contaminant data.

Birth cohort	Full name and key reference	Country	Regions covered	Enrollment period	No. of children at birth
Eligible cohorts that completed the ENRIECO inventory questionnaires:										
ABCD		Amsterdam Born Children and their Development study (van Eijsden et al. 2010)		Netherlands		Amsterdam		2003–2004		7,863
ALSPAC		The Avon Longitudinal Study of Parents and Children (Golding et al. 2001)		United Kingdom		Bristol		1991–1992		14,062
ArcRisk-Norway		Impacts on health in the Arctic and Europe owing to climate-induced changes in contaminant cycling		Norway		Troms, Finnmark, and Nordland		2007–2009		430
BAMSE		The Stockholm Children Allergy and Environmental Prospective Birth Cohort Study (Wickman et al. 2002)		Sweden		Stockholm		1994–1996		4,089
BiB		Born in Bradford (Raynor 2008)		United Kingdom		Bradford		2007–2010		13,000
Co.N.ER		Cohort of newborns in Emilia Romagna (Porta et al. 2006)		Italy		Bologna		2004–2005		654
Czech		Czech Republic Early Childhood Health (Dejmek et al. 2000)		Czech Republic		Teplice and Prachatice		1994–1999		7,577
DARC		The Danish Allergy Research Centre cohort (Johnke et al. 2005)		Denmark		Odense		1998–1999		562
DNBC		Danish National Birth Cohort (Olsen et al. 2001)		Denmark		Denmark		1996–2002		96,986
Duisburg		Duisburg cohort (Wilhelm et al. 2008)		Germany		Duisburg		2000–2003		234
EDEN		Study of determinants of pre and postnatal developmental, psychomotor development and child health (Drouillet et al. 2009)		France		Nancy, Poitiers		2003–2006		1,899
ELFE		French longitudinal study of children (Vandentorren et al. 2009)		France		France		2011–2012		20,000
Faroes*a*		Children’s Health and the Environment in the Faroes (Grandjean et al. 1992, 1997)		Faroe Islands		Faroe Islands		1986–2009		2,351
FLEHS I		Flemish Environment and Health Survey (Koppen et al. 2009)		Belgium		Flanders		2002–2004		1,196
GASPII		Gene and Environment: Prospective Study on Infancy in Italy (Porta et al. 2007)		Italy		Rome		2003–2004		708
Generation R		Generation R (Jaddoe et al. 2008)		Netherlands		Rotterdam		2001–2006		9,778
Generation XXI		Generation XXI (Pinto et al. 2009)		Portugal		Porto		2005–2006		8,647
GINIplus		German Infant Nutritional Intervention Study – plus influence of pollution and genetics on allergy development (Zirngibl et al. 2002)		Germany		Munich, Wesel		1995–1998		5,991
HUMIS		Norwegian Human Milk Study (Eggesbo et al. 2009)		Norway		Norway		2002–2011		2,500
INMA old*b*		Environment and Childhood (Guxens et al. 2011)		Spain		Granada, Menorca, Ribera d’Ebre		1997–2002		1,252
INMA new*c*		Environment and Childhood (Guxens et al. 2011)		Spain		Asturias, Gipuzkoa, Sabadell, Valencia		2003–2008		2,505
INUENDO*d*		Biopersistent organochlorines in diet and human fertility (Toft et al. 2005)		Greenland, Sweden, Poland, Ukraine		Greenland, Sweden (east and west coast), Warsaw (Poland), Kharkiv (Ukraine)		2002–2004		1,322
KANC		Kaunas cohort (Grazuleviciene et al. 2009)		Lithuania		Kaunas		2007–2009		4,000
KOALA		Child, parents and health: lifestyle and genetic constitution (Kummeling et al. 2005)		Netherlands		Southern Netherlands		2000–2003		2,834
Kraków		Kraków cohort (Jedrychowski et al. 2003)		Poland		Kraków		2000–2003		505
Leicester*e*		The Leicester Respiratory Cohorts (Kuehni et al. 2007)		United Kingdom		Leicestershire and Rutland		1985–1997		10,350
LISAplus		Influences of life-style related factors on the immune system and the development of allergies in childhood – plus the influence of traffic emissions and genetic (Heinrich et al. 2002)		Germany		Munich, Wesel, Leipzig, Bad Honneff		1997–1998		3,097
LUKAS		LUKAS (Karvonen et al. 2009)		Finland		Kuopio, Jyväskylä, Joensuu, and Iisalmi		2002–2005		442
MAS		Multicentre Allergy Study (Bergmann et al. 1994)		Germany		Berlin, Duesseldorf, Freiburg, Mainz, Munich		1990		1,314
MoBa		The Norwegian Mother and Child Cohort Study (Magnus et al. 2006)		Norway		Norway		1999–2008		107,000
NINFEA		Birth and Infancy: Effects of the Environment (Richiardi et al. 2007)		Italy		Italy		2005–		7,500
PARIS		Pollution and Asthma Risk: An Infant Study (Clarisse et al. 2007)		France		Paris		2003–2006		3,840
PCB cohort		Early Childhood Development and PCB exposures in Slovakia (Sonneborn et al. 2008)		Slovakia		Michalovce, Stropkov, Svidnik		2001–2003		1,134
PÉLAGIE		Endocrine disruptors: longitudinal study on pregnancy abnormalities, infertility, and childhood (Chevrier et al. 2011)		France		Brittany		2002–2006		3,421
PIAMA		Prevention and Incidence of Asthma and Mite Allergy (Brunekreef et al. 2002)		Netherlands		Northern, western and central parts		1996–1997		3,963
REPRO_PL		Polish Mother and Child Cohort (Polanska et al. 2009)		Poland		Lodz, Wroclaw, Lask, Kielce, Katowice, Legnica, Lublin, Szczecin, Piekary Slaskie		2007–2011		1,800
RHEA		Mother Child Cohort in Crete (Vardavas et al. 2010)		Greece		Heraklion, Crete		2007–2008		1,500
Potentially eligible cohorts that did not complete the ENRIECO inventory questionnaire:										
Ukraine		Children of Ukraine (Hryhorczuk et al. 2009)		Ukraine		Kyiv, Dniprodzerzhynsk, Mariupol		1992–1996		4,510
MAAS		The National Asthma Campaign Manchester Asthma and Allergy Study (Custovic et al. 2002)		United Kingdom		Manchester		1995–1997		1,211
Northern Adriatic Cohort		Northern Adriatic Cohort (Barbone et al. 2004)		Italy		Northeastern Italy		1999–2001		243
**a**The Faroes cohorts consist of four subcohorts: cohort 1 (enrollment: 1986–1987; *n* = 1022), cohort 2 (enrollment: 1994–1995; *n* = 182), cohort 3 (enrollment: 1997–2000, *n* = 656), cohort 5 (enrollment: 2007–2009; *n* = 491); **b**INMA old consists of three subcohorts: Granada (enrollment: 2000–2002; *n* = 668), Menorca (enrollment: 1997–1998; *n* = 482), Ribera Ebre (enrollment: 1997–1999; *n* = 102); **c**INMA new consists of four subcohorts: Asturias (enrollment: 2004–2007; *n* = 485), Gipuzkoa (enrollment: 2006–2008; *n* = 611), Sabadell (enrollment: 2004–2007; *n* = 622), Valencia (enrollment: 2003–2005; *n* = 787); **d**INUENDO consists of four subcohorts: Greenland, Sweden (east and west coast), Warsaw (Poland), Kharkiv (Ukraine); **e**The Leicester Respiratory Cohorts consist of two subcohorts: the Leicester I cohort (enrollment: 1985–1989; *n* = 1,650) and the Leicester II cohorts (enrollment: 1993–1997; *n* = 8,700).										

**Figure 2:**
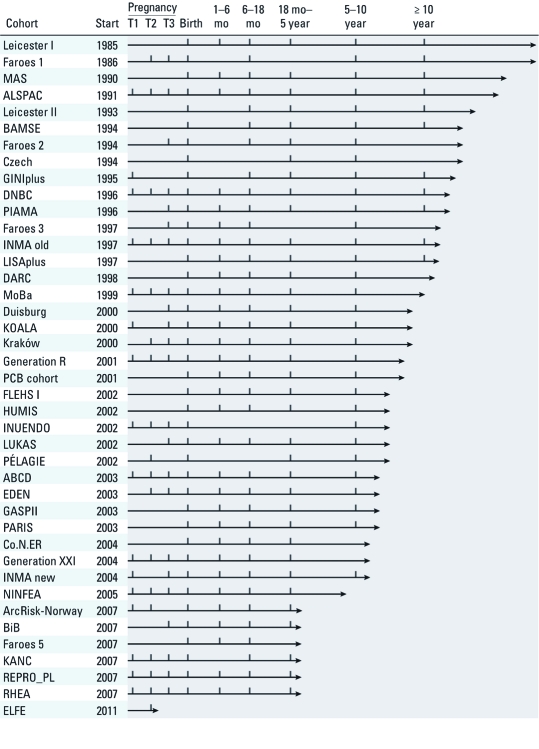
Start of enrollment and time points of follow-up*^a^* (vertical bars) in European birth cohorts collaborating in ENRIECO. T1, T2, and T3 are first, second, and third trimesters. mo, months. See Table 1 for full names and locations of cohorts. *^a^*These are points in predefined periods. Some cohorts have many more (e.g., yearly) follow-points not reflected in this figure. The four Faroes cohorts and the two Leicester cohorts are shown separately because they have different time lines.

At our reference date (February 2011), children in 19 cohorts were between 5 and 10 years of age, children in 12 cohorts were > 10 years of age, and in five cohorts they were < 5 years of age. One cohort in France, Etude Longitudinale Française depuis l’Enfance (ELFE), started data collection in April 2011. Most of the cohorts started recruitment of mothers during pregnancy (*n* = 25); the remaining started at birth. Most cohorts have multiple follow-up points after birth, and the majority have follow-up points in each of the child age periods specified in the questionnaire (1–6 months, 6–18 months, 18 months–5 years, 5–10 years, > 10 years) (Figure 2).

Biological samples were collected in all cohorts from the mother and/or the child and, more rarely, from the father (Table 2). Sampling covered prenatal and postnatal periods and many types of tissue, including blood, urine, hair, nails, breast milk, placentas, and saliva. All but six cohorts [The Stockholm Children Allergy and Environmental Prospective Birth Cohort Study (BAMSE); German Infant Nutritional Intervention Study—plus influence of pollution and genetics on allergy development (GINIplus); The Leicester Respiratory Cohorts (Leicester); Birth and Infancy: Effects of the Environment (NINFEA); Pollution and Asthma Risk: An Infant Study (PARIS); Prevention and Incidence of Asthma and Mite Allergy (PIAMA)] collected, or plan to collect, blood samples from the mother during pregnancy or cord blood at birth.

**Table 2:** Biological samples collected in the European birth cohorts collaborating in ENRIECO.*a*

Mother					Child						Father	
					Birth	1–6 months	7–18 months	19 months–5 years	5–10 years	≥ 10 years		
Cohort	T1	T2	T3	Birth							Postnatal	Prenatal	Postnatal
ABCD	B									B			
ALSPAC	B, U	B, U	B, U	P	H, N	CB		H, N	B, H, N	B, U, T	B, U, S		H, N
ArcRisk-Norway		B, U		B, U, H	B, U, BM	CB, B, M							
BAMSE									B, U	B			
BiB			B, U			CB		B	B			S	
Co.N.ER				B		CB							
Czech				B, P		CB			U	U			
DARC						CB	B	B	B	B			
DNBC	B	B				CB	B						
Duisburg			B		B, U, BM	CB			B, U	B, U			
EDEN		B, U, S		H, BM, P		CB, H, M		H	H	B, U, H		B	
ELFE				B, U, BM, H		CB, CT, M			U, H, S	U			
Faroes			B	H, BM	B, U, H, BM	CB	B	B, H	B, H	B, H	B, H		U, H, Se
FLEHS I					S	CB	F	F	U	U			
GASPII				B		CB							
Generation R	B, U	B, U	B, U			CB			S	B, U, S		B	
Generation XXI	B	B	B, AT		B	CB			B				B
GINIplus									B	B	B		
HUMIS*b*		B, U		B	BM	CB				S		B	
INMA old	B	B	B	B, BM, P	B, BM	CB, U	B	B	B, U, H, S	B, U		B	
INMA new	B, U		U, N	BM, P	U, S	CB, H		U, N, S	H, S	B, U, H, S			B, S
INUENDO	B	B	B							S		B, Se	
KANC			B			B							
KOALA			B		BM, S		F	S	B	B, F			S
Kraków				B, P	U	CB, M			U	B, U			U
Leicester											S		
LISAplus						CB, B			B	B	B		
LUKAS				B, P	BM	CB	F	B, F	B	B, U			B
MAS				B		CB, B		B, U	B, U	B, U	B		B
MoBa		B, U		B		CB						B	
NINFEA					S		S						
PARIS									B	B, U			
PCB cohort				B, BM		CB	B	B	B	B, U			
PELAGIE	U			H, P		CB				S, U			
PIAMA					B, BM, S		B	B	B, S	B	B, S		B, S
REPRO_PL	B, S	B, U, S	B, U, H, S	B	BM	CB		B, U	B, U				
RHEA	B, U	U		B		CB		U, H	B				B, Se
Abbreviations: AT, adipose tissue; B, blood; BM, breast milk; CB, cord blood; CT, cord tissue; F, feces; H, hair; M, meconium; N, nails; P, placenta; S, saliva or buccal swabs; Se, semen; T, teeth; U, urine. T1, T2, and T3 are first, second, and third trimesters. See Table 1 for full names and locations of cohorts. **a**Many but not all ENRIECO birth cohorts are also included in the BBMRI Catalogue of European Biobanks (BBMRI 2011), which can be queried by specimen type. **b**Blood, urine, and cord blood are available through overlap with MoBa in approximately 800 participants.													

Many cohort studies collected information on passive smoking (*n* = 36), maternal occupation (*n* = 33), outdoor air pollution (*n* = 27), and allergens/biological organisms (*n* = 27) (Table 3). Fewer cohorts have collected information on water contamination (*n* = 13), ionizing or nonionizing radiation exposures (*n* = 12), noise (*n* = 14), or emerging chemical exposures such as phthalates and bisphenol A (*n* = 17). Details of each assessment (methods, numbers of subjects, and time period of assessment) can be found in the Web inventory (ENRIECO 2011). Twenty-seven cohorts have measured or are measuring contaminants or their metabolites as markers of internal dose, mostly in the categories of metals, persistent organic pollutants, and tobacco smoke (Table 4).

**Table 3:** Assessment of environmental exposures (prenatal or postnatal) in European birth cohorts participating in ENRIECO.*a*

Cohort	Air pollution outdoor	Air pollution indoor	Water contamination	Allergens and biological organisms	Metals*b*	Pesticides*c*	Persistent organic pollutants	Other chemical exposures*d*	Radiations	Passive smoking*e*	Noise	Occupation
ABCD	X					X			X*g*	X		X
ALSPAC		X		X	X	X	X*g*		X	X	X	X
ArcRisk-Norway					X		X	X*g*		X		X
BAMSE	X	X		X						X		X
BiB	X		X									X
Co.N.ER	X*g*	X		X						X	X	X
Czech	X									X		X
DARC	X	X		X						X		X
DNBC	X*g*	X		X		X	X		X	X		X
Duisburg	X	X	X*g*	X	X		X	X		X		X
EDEN	X	X	X*g*		X			X	X	X		X
ELFE	X*g*	X*g*	X*g*	X*g*	X*g*	X*g*	X*g*	X*g*	X*g*	X*g*		X*g*
Faroes					X	X*g*	X	X*g*		X		X
FLEHS I	X	X		X	X	X	X			X		X
GASPII	X	X		X						X	X	
Generation R	X			X		X	X*g*	X		X	X	X
Generation XXI				X						X		X
GINIplus	X	X		X						X		
HUMIS*f*	X	X	X	X	X*g*	X	X	X	X	X		X
INMA old	X	X	X	X	X*g*	X	X	X	X	X	X	X
INMA new	X	X	X	X	X	X	X	X	X	X	X	X
INUENDO					X*g*	X*g*	X*g*	X*g*		X		X
KANC	X		X							X	X*g*	X
KOALA		X	X	X					X*g*	X	X	X
Kraków	X	X		X	X	X	X	X		X		X
Leicester	X	X		X						X		X
LISAplus	X	X		X						X	X*g*	
LUKAS		X		X	X	X	X	X*g*		X		X
MAS		X		X						X	X	X
MoBa	X*g*	X	X	X	X*g*	X	X*g*	X	X	X	X	X
NINFEA	X*g*	X*g*		X*g*		X*g*		X*g*	X*g*	X*g*	X*g*	X*g*
PARIS	X	X	X	X						X		X
PCB cohort		X			X	X	X	X*g*		X		X
PELAGIE		X	X		X	X	X	X		X		X
PIAMA	X	X		X						X		
REPRO_PL	X*g*			X	X		X			X	X	X
RHEA	X	X	X	X	X	X	X*g*	X*g*	X	X	X	X
See Table 1 for full names and locations of cohorts.**a**Details of each assessment (method, time period, number of subjects) are available at www.birthcohortsenrieco.net (ENRIECO 2011). **b**Biomonitoring data only, not questionnaires. **c**Organochlorine pesticides are included under persistent organic pollutants. **d**Phthalates and phenols (including bisphenol A). **e**Passive smoking exposure of the mother during pregnancy or of the child postnatally. **f**Data on occupation and passive smoking are available through overlap with MoBa in approximately 800 participants. **g**Measurements are planned or ongoing but not completed.												

**Table 4:** Biomarkers of selected environmental exposures measured by European birth cohorts collaborating in ENRIECO.*a*

Cohort	Metals	Persistent organic pollutants	Other pesticides	Tobacco smoking	Other chemicals
ALSPAC		As, Cd, Hg, Mn, Pb, Se,*b* TMS		PFCs*b*		—		Cotinine		—
ArcRisk-Norway		As, Cd, Co, Hg, Mb, Mn , Pb		Chlordane, DDT/DDE, HCB, PCBs		—		—		Planned
BAMSE		—		—		—		Cotinine		—
Czech		—		—		—		Cotinine		PAHs
DNBC		—		PFCs		—		—		—
Duisburg		Cd, Hg, Pb, Se		DDT/DDE, HCB,*b* HCH, PCBs, PCDDs, PCDFs, PFCs		—		Cotinine*b*		BPA, phthalates
EDEN		B, Cd, Mn, Hg, Pb		—		—		Cotinine		Phthalates, phenols (including BPA)
ELFE		Al,*b* As,*b* Cd,*b* Hg,*b* Pb*b*		BFRs,*b* organochlorines,*b* PFCs,*b* PCBs,*b* PCDDs,*b* PCDFs*b*		Organophosphates,*b* pyrethroids*b*		Cotinine*b*		BPA,*b* phthalates*b*
Faroes		Hg, Pb, Se		BFRs,*b* chlordane, DDT/DDE, dieldrin/endrin, heptachlor, HCB, β-HCH, mirex, organometallic compounds, PCBs, PFCs,*b* toxaphene		—		—		BPA,*b* phthalates*b*
FLEHS I		Cd, Pb		DDT/DDE, dioxin-like compounds, HCB, PCBs		—		—		—
Generation R		—		Organochlorines*b*		Organophosphates		—		BPA, phthalates
GINIplus		—		—		—		Cotinine		—
HUMIS		Hg,*b* Pb*b*		BFRs, DDT/DDE, HCB, HCH, mirex, PCBs, PCDDs, PCDFs, PFCs, toxaphene		—		—		Phthalates
INMA old		Hg, Pb,*b* TMS*b*		Aldrin/dieldrin/endrin, BFRs, DDT/DDE, endosulfan, lindane, methoxychlor, mirex, HCB, HCH, PCBs		—		Cotinine		Phthalates, phenols (including BPA)
INMA new		Hg, Pb, TMS*b*		BFRs, DDT/DDE, HCB, HCH, PCBs		—		Cotinine		Phthalates, phenols (including BPA)
INUENDO		Cd,*b* Hg,*b* Pb*b*		BFRs,*b* DDT/DDE, HCB,*b* PCBs, PFCs*b*		—		—		BPA,*b* phthalates*b*
Kraków		Cd, Hg, Pb		—		—		Cotinine		Phthalates, PAHs, benzo[a]pyrene-adducts
LISAplus		—		—		—		Cotinine		—
LUKAS		As, Cd, Hg, Pb, Se		BFRs, DDT/DDE, organometallic compounds, PCBs, PCDDs, PCDFs, polychlorinated naphthalene		—		—		Phthalates*b*
MAS		—		—		—		Cotinine		—
MoBa		Planned		BFRs,*b* DDT/DDE,*b* PCBs*b*		Organophosphates		—		BPA, phthalates
NINFEA		—		—		—		—		—
PCB cohort		Hg, Pb		DDT/DDE, HCB, HCH, PCBs, PFCs*b*		—		—		Phthalates*b*
PÉLAGIE		Hg		Aldrin, BFRs, DDT/DDE, dieldrin/endrin, heptachlor, HCB, PCBs		Acetochlor, alachlor, metolachlor, organophosphorus, propoxur, triazines		—		Phthalates
REPRO_PL		Cd, Hg, Pb, Se, Zn, Cu		PCBs, PCDDs, PCDFS		—		Cotinine		PAHs (1-hydroxypyrene)
RHEA		As, Cd, Hg, Mn, Pb		DDT/DDE,*b* HCB,*b* PCBs,*b* PCDDs,*b* PFCs*b*		—		NNAL, cotinine		Phthalates*b*
Abbreviations: As, arsenic; B, boron; BFRs, brominated flame retardants; BPA, bisphenol A; Cd, cadmium; Co, cobalt; Cu, copper; DDE, dichlorodiphenyldichloroethylene; DDT, dichlorodiphenyltrichloroethane; HCB, hexachlorobenzene; HCH, hexachlorocyclohexane; Hg, mercury; Mb, molybdenum; Mn, manganese; Ni, nickel; NNAL, 4-(methylnitrosamino)-1-(3-pyridyl)-1-butanol; PAHs, polycyclic aromatic hydrocarbons; Pb, lead; PCBs, polychlorinated biphenyls; PCDDs, polychlorinated dibenzo-*p*-dioxins; PCDFs, polychlorinated dibenzo furans; PFCs, perfluorinated compounds; Se, selenium; Tl, thallium; TMS, total metals spectrum; Zn, zinc. See Table 1 for full names and locations of cohorts. **a**Includes all exposures biomarkers measured as part of the exposure topics included the inventory questionnaire [see Supplemental Material, Annex 1 (http://dx.doi.org/10.1289/ehp.1103823)]. Details of each assessment (method, time period, number of subjects) are available at www.birthcohortsenrieco.net (ENRIECO 2011).** b**Measurements are planned or ongoing but not completed.										

All cohorts have information on birth outcomes, mostly birth weight, gestational age, and mode of delivery [see Supplemental Material, Annex 2 (http://dx.doi.org/10.1289/ehp.1103823)]. Fewer asked questions about time to pregnancy or collected data on congenital anomalies. Fifteen cohorts collected information on ultrasound measurements. Neurodevelopment and behavioral development of the children was assessed in 26 cohorts, using a large variety of methods including questionnaires, neuropsychological tests, and school results. Nineteen cohorts assessed cognitive function of the children (e.g., global IQ, executive function, memory, etc.), 18 cohorts assessed behavior or ADHD (attention deficit/hyperactivity disorder) symptoms, and few have data on the clinical diagnosis of autism (*n* = 13) or mental health problems (*n* = 3). Names of the questionnaires and tests used to assess neurodevelopment, as well as the developmental periods at which they were used are available in the Web inventory. Growth and obesity and allergies (*n* = 34), and asthma and respiratory infections (*n* = 33) were assessed in nearly all cohorts. Metabolic syndrome indicators, such as blood pressure or cholesterol levels, were collected in around half of the cohorts. Childhood cancer (*n* = 12) and sexual maturation (*n* = 14) have been assessed in few cohorts to date [see Supplemental Material, Annex 2 (http://dx.doi.org/10.1289/ehp.1103823)].

## Discussion

More than 35 birth cohorts in Europe, studying more than 350,000 mother–child pairs, have data that can be used to examine associations between chemical, physical, and biological environmental exposures and child health. The searchable Web-based inventory is the first place where this information is made publicly available. The inventory intends to facilitate collaborations between birth cohort researchers in this field and can be searched to identify cohorts relevant for comparison, replication, and combined studies. Moreover, it may be used by policy makers and other stakeholders to identify birth cohorts that can provide specific information on environmental exposures or related outcomes. This initiative may quite easily be expanded beyond Europe to stimulate collaboration between cohorts worldwide.

The cohorts we evaluated include a few very large general cohorts with multiple aims as well as many smaller cohorts, which are generally more focused on specific environmental risk factors or specific health outcomes. The distribution of the identified birth cohorts is not homogeneous across Europe, with more and larger cohorts mostly located in the north and west of Europe and fewer and smaller cohorts in the south and east. Indeed, more than half of all mother–child pairs come from two very large cohorts in Norway and Denmark. However, studies of environmental contaminant exposures, especially those measuring exposure biomarkers, usually cannot cover large numbers of subjects. Smaller, specialized cohorts, often located in high-exposure areas, have made very important contributions in this regard.

Passive smoking, maternal (and paternal, less often) occupation, air pollution, and allergen exposures have been assessed by many birth cohorts. Efforts to combine data analyses on these topics are already ongoing within international projects such as European Study of Cohorts for Air Pollution Effects (ESCAPE) (2009), Global Allergy and Asthma European Network (GA2LEN) (2009), and International Collaboration on Air Pollution and Pregnancy Outcomes (ICAPPO) (Woodruff et al. 2010). Biomarker data on some metals (lead and mercury) and some persistent organic pollutants [polychlorinated biphenyls (PCBs) and dichlorodiphenyldichloroethylene (DDE)] are also available in a relatively large number of cohorts, but usually for a small number of subjects in each cohort and with considerable variation in the type and timing of the biological samples. Studies combining data from different cohorts have been conducted for few such pollutants so far; examples are the 7-cohort lead meta-analysis (Lanphear et al. 2005) and a recent meta-analysis of 12 European cohorts evaluating PCB and DDE exposure and fetal growth (Govarts et al. 2011). Exposure areas that are less well covered in the cohorts include noise, ionizing and nonionizing radiations, and chemical exposures such as brominated and fluorinated compounds, phthalates, and phenols (including bisphenol A). These are areas of emerging concern for child health and high on international research agendas [e.g., the World Health Organization (WHO) Research Agenda for Radiofrequency Fields] (WHO 2010). Although actual measurements are scarce, future assessments of these exposures are planned in many cohorts, making this an optimal time to work toward a high level of data comparability.

Birth cohorts are most suitable to examine child health outcomes that are common or can be measured on a continuous scale. Rare outcomes and pathologies such as extremely preterm birth, childhood cancers, congenital anomalies, and autism can be studied only in the very large cohorts or by combining cohorts, as is being done, for example, in the International Childhood Cancer Cohort Consortium (Brown et al. 2007). All cohorts collected information on fetal growth and gestational length and on childhood anthropometric measures; combined studies of these outcomes appear feasible. A number of cohorts specialize in the early causes of asthma and allergies, and combined studies of these outcomes are underway (Keil et al. 2006a, 2006b). Neurodevelopment is a wide area that encompasses many cognitive and behavioral outcomes and includes even more assessment instruments, ranging from parental questionnaires on developmental milestones to detailed neuropsychological assessments. Combined analyses may therefore prove difficult and may need to be restricted to cohorts that use similar tests in similar age groups.

This inventory inevitably simplifies the data available in the cohorts. For example, we summarized time periods over which data were collected, meaning that not each follow-up point in each cohort is represented. Furthermore, we restricted information about exposure and health outcome assessment methods to relatively basic questions [see Supplemental Material, Annex 1 (http://dx.doi.org/10.1289/ehp.1103823)]. It is also inevitable that the inventory information will become out of date quickly. The Web-based database will be maintained by sending requests for updates to the included and newly identified cohorts at approximately yearly intervals for as long as resources allow. The inventory is meant to give researchers and other professionals looking for specific data the first overview of relevant cohorts; only direct contact with the cohorts can then establish the suitability of data for a specific purpose. Beyond our overview here, the ENRIECO project has made an extensive effort to compare available data and methods across cohorts and to develop recommendations for the use of existing data and for the collection of data in the future. These reports are available on request (ENRIECO 2009).

The 37 European birth cohorts described in this review fulfilled all our inclusion criteria. We also identified other studies that did not strictly match our criteria of a birth cohort study or did not collect data on the environmental exposures. We may have missed other birth cohorts that have not yet been published or are still in planning stages. In addition, many important birth cohorts outside Europe were not included. The Web-based inventory will be open for the inclusion of additional birth cohorts for the foreseeable future. Overall, we have achieved an excellent response from the European cohorts; only three potentially eligible cohorts did not return questionnaires. Involvement of the cohorts in workshops, working groups, and case studies was instrumental in these results.

*Future collaboration.* The 350,000 mother–​child pairs covered by European birth cohorts form an impressive number, but of course this is only a small percentage of all births in Europe [(> 5 million births annually in 25 EU countries (Eurostat 2011)]. For the investigation of any specific exposure–outcome relationship, the number of available subjects will be much smaller than the total number of children followed by the cohorts. The cohorts are in very different stages of follow-up and thus have not all reached the ages at which some outcomes, for example, asthma or puberty onset, can be assessed. Moreover, not all exposure and outcome topics included in ENRIECO are immediately suitable for combined analyses of individual data. However, there are many reasons why continued and tightly coordinated collaboration between existing cohorts is desirable (Kogevinas et al. 2004; Willett et al. 2007): *a*) combined analyses of cohort data to improve statistical power and reduce publication bias; *b*) improved contrast and diversity in exposure and outcome; *c*) faster response to concerns about new environmental exposures; *d*) facilitation of replication and comparison studies in different settings; *e*) facilitation of linkage to large-scale, routinely collected environmental and health data; and *f*) widening expertise to improve methodological approaches, including protocols of biological and environmental sample collection and analysis.

In conclusion, the impressive resource of existing data in birth cohorts should form the basis for a long-term infrastructure that brings together birth cohort research on early-life environmental contaminant exposure and child health, with the ultimate aim to improve causal inference in this field. Further expansion to countries outside Europe should be one important aim for the future. This will be possible only with continued support for coordination.

## Supplemental Material

(147 KB) PDF
