# Supplementary material for: European Birth Cohorts for Environmental Health Research
Source: Environ Health Perspect. 2011 Aug 29;120(1):29–37. doi: 10.1289/ehp.1103823 (PMC3261945; doi:10.1289/ehp.1103823)
Supplement: (147 KB) PDF [file ehp.1103823.s001.pdf]

Supplemental Material

European Birth Cohorts for Environmental Health Research

Authors

Martine Vrijheid, Maribel Casas, Anna Bergström, Amanda Carmichael, Sylvaine Cordier, Merete Eggesbø, Esben Eller, Maria P Fantini, Marieta F Fernández, Ana Fernández-Somoano, Ulrike Gehring, Regina Grazuleviciene, Cynthia Hohmann, Anne M Karvonen, Thomas Keil, Manolis Kogevinas, Gudrun Koppen, Ursula Krämer, Claudia E Kuehni, Per Magnus, Renata Majewska, Anne Marie Nybo Andersen, Evridiki Patelarou, Maria Skaalum Petersen, Frank H Pierik, Kinga Polanska, Daniela Porta, Lorenzo Richiardi, Ana Cristina Santos, Rémy Slama, Radim J Sram, Carel Thijs, Christina Tischer, Gunnar Toft, Tomáš Trnovec, Stephanie Vandentorren, Tanja GM Vrijkotte, Michael Wilhelm, John Wright, Mark Nieuwenhuijsen

Table of Contents

Annex 1 – ENRIECO Inventory Questionnaire…………………………………….........Page 2

Annex 2 – Assessment of birth outcomes in European birth cohorts participating in ENRIECO

Table 1 – Reproductive and birth outcomes ………....……………………….. Page 30

Table 2 – Neurodevelopment outcomes……………………………………...Page 31

Table 3 – Asthma, allergy, and respiratory outcomes, cancer, growth and obesity, metabolic syndrome, sexual maturation.………………..………………………Page 32

Annex 1: ENRIECO Inventory Questionnaire

A. Basic Protocol Description (update of www.birthcohorts.net)

A1. Identification

- **Cohort name:**

- **Principal investigator:**

- **Contact(s) for environmental exposures:**

- **Cohort website:**

- **Key publication(s) of cohort protocol/methods/description:**

A2. Basic Description

- **Main aim/objectives/focus of cohort:**

- **Source population**

☐ nation-based
☐ region-based
☐ hospital-based
☐ selected (high-risk, exposure etc.), describe:
☐ other:

- **Geographical coverage**, please describe: ___________________

- **Calendar period of enrolment –** calendar years of start and finish: ______________

- **Enrolment - status:**

☐ completed
☐ ongoing
☐ planned

- **Developmental period of enrolment –** give developmental period of start of enrolment:

☐ pre-pregnancy
☐ pregnancy, give pregnancy week(s) ________ weeks of pregnancy
☐ at birth
☐ postnatal, give month(s) ________ months of age

- **Enrolment criteria**, please describe in and exclusion criteria:

- **Expected number of participants** at enrolment when enrolment completed:

_____ mothers _____ fathers _____ children

- **Expected duration of follow-up**: _______ years

A3. Basic Data Collection Scheme

**Type of data collection** (Give estimate of number of subjects for each period where data/samples are available: N=XXX)	**Pregnancy**			**Birth**	**Post natal**				
	1st trimester	2nd trimester	3rd trimester		0-6 months	7-18 months	18-60 months	5-10 years	10+ years
**Questionnaires:**									
maternal exposures									
paternal exposures									
offspring exposures									
maternal outcomes									
paternal outcomes									
offspring outcomes									
**Biological samples:**									
maternal blood									
paternal blood									
cord blood									
offspring blood									
maternal urine									
paternal urine									
offspring urine									
maternal other (hair, nails, saliva, breast milk, etc.)									
Paternal other (hair, nails, saliva etc.)									
offspring other (hair, nails, saliva, etc.)									

B. Exposure Assessment

B1. Outdoor Air Pollution

- **Were *outdoor* air pollution exposures assessed for the members of your cohort?**

☐ yes
☐ not yet, but planned. Please give predicted year of completion: 20______
☐ no, please proceed to part B2

- **Which air pollutants were/will be assessed** *(multiple answers possible)***?**

☐ nitrogen dioxide (NO_2)
☐ nitrogen oxides (NO_x)
☐ particulate matter < 10 µg in diameter (PM_{10})
☐ particulate matter < 2.5 µg in diameter ($PM_{2.5}$)
☐ soot content of particulate matter
☐ ozone (O_3)
☐ others: ________________________

- **Which types of exposure assessment were/will be used** *(multiple answers possible)***?**

☐ dispersion modelling
☐ land-use regression modelling
☐ routine air monitoring network measurements (e.g. nearest monitor, interpolation)
☐ exposure indicator variables (e.g. traffic density; proximity to traffic/industry from geographic information system)
☐ questionnaires (e.g. self-reported traffic densities; proximity to traffic or industrial sources; wood smoke exposure). Please describe: ______________________
☐ individual measurements (e.g. personal monitoring, stationary measurements outside/inside participants' homes). Please describe:___________________

- **Details of the assessments (complete table with pollutant, assessment method, timing, and rough number of assessments):**

**Type of air pollution assessment**		**Pregnancy (mother)**			**Post natal (child)**	Number/% of the cohort
Air pollutant	Method	1st trim.	2nd trim.	3rd trim.	Age	
Completed:						
Planned:						

B2. Indoor Contaminants (for example from combustion sources, cleaning products, or any indoor exposures not already included in other sections)

- **Were exposures to *indoor* contaminants assessed for the members of your cohort?**

☐ yes
☐ not yet, but planned. Please give predicted year of completion: 20______
☐ no (proceed to part B3)

- **Which indoor contaminants were/will be assessed?**

☐ nitrogen dioxide (NO_2)
☐ VOCs
☐ gas cooking / heating / appliances
☐ cleaning products
☐ others:

- **Which types of exposure assessment were/will be used (*multiple answers possible*)?**

☐ modelling
☐ individual measurements (e.g. personal monitoring, indoor air monitoring)
☐ questionnaires (e.g. use of gas cookers, type of heating system, use of cleaning products)
☐ other:

- **Details of the assessments (complete table with pollutant/contaminant, assessment method, timing, and rough number of assessments):**

**Type of indoor contaminant assessment**		**Pregnancy (mother)**			**Post natal (child)**	Number/% of the cohort
contaminant	Method	1st trim.	2nd trim.	3rd trim.	Age	
Completed:						
Planned:						

Further description:

B3. Water Contamination

- **Were water contaminants assessed for the members of your cohort?**

☐ yes
☐ not yet, but planned. Please give predicted year of completion: 20_____
☐ no (proceed to part B4)

- **Which water contaminants were/will be assessed?**

☐ disinfection by products
☐ pesticides
☐ metals
☐ endocrine disrupting substances
☐ others: ____

- **Which type of assessment/questionnaire questions are/were used?**

☐ routine monitoring water concentration data from water companies or government
☐ newly collected water concentration data (measurements)
☐ questionnaire:
  ☐ information on tap/bottled water ingestion
  ☐ information on coffee, tea and other tap water based beverage ingestion
  ☐ information on swimming habits
  ☐ information on showering/bathing habits
☐ personal biomonitoring
☐ other:

- **Details of the assessments (complete table with pollutant/contaminant, assessment method, timing, and rough number of assessments):**

**Type of water contaminant assessment**		**Pregnancy (mother)**			**Post natal (child)**	Number/% of the cohort
contaminant	Method	1st trim.	2nd trim.	3rd trim.	Age	
Completed						
Planned:						

Further description:

B4. Allergens and biological organisms

- **Were exposures to allergens and biological organisms assessed for the members of your cohort?**

☐ yes
☐ not yet, but planned. Please give predicted year of completion: 20____
☐ no (proceed to part B5)

- **Which allergens and biological organisms <u>were assessed or will be assessed</u>** *(indicate whether completed or planned)***?**

**Type of allergens**	**Assessment method**		**Timing of the assessment (please specify)**			**% or N of the cohort measured**
	Direct measurement from house dust or air samples	Surrogate* (please specify)	During pregnancy	0-1 years (up to 12 months)	During Early Childhood	
Mite- Der p						
Der f						
Pets- Cat						
Dog						
Other pets (please specify)						
Mold-Cladosporium						
Penicillium						
Aspergilus						
Alternaria						
Other mold allergens						
Cockroach						
Pollen						
Other						
**Other bio-contaminant:**						
Pests & Vermin						
Endotoxin						
Mold-Eps						
Glucan						
Other mold species						
Other						

* Please specify the surrogate measures, for example: cat ownership for cat allergen, humidity for mold exposure in general, spore counts for mold allergen, season of birth for specific pollen.

B5. Heavy Metals

- **Was exposure to heavy metals assessed for the members of your cohort?**

☐ yes
☐ not yet, but planned. Please give predicted year of completion: 20_____
☐ no (please proceed to part B6)

- **Which agents were/will be assessed?**

☐ mercury (Hg)
☐ lead (Pb)
☐ cadmium (Cd)
☐ arsenic (As)
☐ manganese (Mn)
☐ total metals spectrum
☐ other: ____________________________________

- **Which type of assessment was/will be used?**

☐ biological sampling
☐ environmental sampling
☐ questionnaires
☐ occupational exposure / JEM
☐ dietary exposure (please specify: ______________________)
☐ through tap water consumption

- **Details of the biomonitoring analyses (complete table with heavy metal, medium, person, timing, and rough number analysed):**

**Type of heavy metal assessment**		**Pregnancy (mother)**			**Birth (mother or child)**	**Post natal (child)**	
Metals	Medium	1st trim.	2nd trim.	3rd trim.		Age	Number
Completed:							
Planned:							

B6. Pesticides

- **Was exposure to pesticides assessed for the members of your cohort?**

☐ yes
☐ not yet, but planned. Please give predicted year of completion: 20________
☐ no (please proceed to part B7)

Assessment of exposure at *individual* level

- **Which type of assessment was/will be used?**

☐ biological sampling
☐ questionnaire data on self-reported pesticide use (in home)
☐ occupational exposure
☐ dietary exposure
☐ environmental survey (dust, etc…)
☐ other: _________________

- **Details of the assessments (complete table with pesticides type, medium, person, timing, and rough number of assessments):**

**Type of pesticide assessment**		**Pregnancy (mother)**			**Birth (mother or child)**	**Post natal (child)**	
Pesticide	Medium	1st trim.	2nd trim.	3rd trim.		Age	Number
Completed:							
Planned:							

Assessment of exposure at a *geographical* level:

- **Which type of assessment was/will be used?**

☐ land/crop data:
geographical scale (national, regional, etc.): _____________________
years available (relevant to your cohort): _____________________
☐ pesticide usages in crops:
official recommendations or real uses: _____________________
geographical scale (national, regional, etc.): _____________________
years available (relevant to your cohort): _____________________
☐ drinking water contamination:
groups of pesticides: _____________________
frequency (yearly, monthly, etc.): _____________________
☐ air measurements:
groups of pesticides: _____________________
frequency (yearly, monthly, etc.): _____________________

B7. Radiations: EMF/UV/ionising

- **Were any of the following sources of non-ionising or ionising radiation exposure assessed for the members of your cohort?**

☐ power lines
☐ mobile phone handsets (use of a mobile phone)
☐ mobile phone base stations
☐ other RF exposures such as WiFi, cordless phones
☐ occupational EMF exposure
☐ sun (sun bathing, sun beds, application of protection creams, etc)
☐ medical ionising radiation exposures (CT scans, X-rays, interventional cardiology procedures)
☐ residential radon exposure
☐ not yet, but planned. Please give predicted year of completion for the specific exposure:
☐ no (proceed to part B8)

- **Which type of assessment was/will be used?** *(copy for each exposure source separately)*

☐ personal monitors
☐ environmental measurements
☐ questionnaire data
☐ geographical data on source location (for power lines, mobile phone base stations, radon)
☐ individual data from mobile phone network operators
☐ occupational exposure / JEM

- **Details of the assessments (complete table with type of assessment, person, timing, and rough number of assessments):**

**Type of assessment**		**Pregnancy (mother)**			**Birth (mother or child)**	**Post natal (child)**	
Exposure	Method	1st trim.	2nd trim.	3rd trim.		Age	Number
Completed:							
Planned:							

B8. Smoking and Second-hand Smoke (SHS)

A. Active smoking of the *mother*

- **Was exposure to *active* tobacco smoke assessed for the members of your cohort?**

☐ yes
☐ not yet, but planned. Please give predicted year of completion: 20________
☐ no (proceed to second-hand smoke)

- **Which type of assessment was used for verifying active smoking (of the mother)?**

☐ biological sampling
☐ questionnaire data (self report)
☐ other:_________________

- **If biomarkers were used to verify active smoking status, if known, what was the cut-off used?**

☐ serum cotinine > _____ ng/ml
☐ urinary cotinine > ______ ng/ml

- **If questionnaires were used, which of the following information is available:**

☐ number of cigarettes smoked per day/week/month
    ☐ at one point during pregnancy
    ☐ at different time points during pregnancy
    ☐ before pregnancy
☐ time (week/month) before conception at which mother quit smoking
☐ time (week/month) during pregnancy at which mother quit smoking
☐ other:_________________

B. Passive smoking / second-hand smoke (SHS)

- **Was exposure to *passive* tobacco smoke / environmental tobacco smoke /second hand smoke (SHS) assessed for the members of you cohort?**

☐ yes
☐ not yet, but planned. Please give predicted year of completion: 20________
☐ no (proceed to part B9)

- **For which members of your cohort did you collect information of exposure to SHS?**

☐ pregnant women
☐ children, age:
☐ other: ____________________

- **Which type of assessment was used for evaluating exposure to SHS (mother/child)?**

☐ biological sampling (i.e cotinine, NNAL)
☐ environmental measurements (nicotine dosimeters, indoor PM 2.5 monitors, dust swipes)
☐ questionnaire data (who smokes at home, at work, visiting smoky places)
☐ other:_________________

- **Details of the active and passive smoking assessments (complete table with type, method, person, timing, and rough number of assessments):**

**Type of smoking assessment**		**Pregnancy (mother)**			**Birth (mother or child)**	**Post natal (child)**	
Compound	Medium	1st trim.	2nd trim.	3rd trim.		Age	Number
Completed:							
NNAL							
Cotinine							
Maternal smoking							
Smoking in home							
Smoking in working place							
Smoking in bars/cafes (before ban of tobacco smoke)							
Planned:							

B9. Noise

- **Was noise exposure assessed for the members of your cohort?**

☐ yes
☐ not yet, but planned. Please give predicted year of completion: 20____
☐ no (proceed to part B10)

A. Objective assessment

- **Was noise data collected by *objective* assessments – noise measurements or noise maps?**

☐ yes
☐ no

- If yes please provide details: ________________________

B. Passive assessment

- **Was noise data collected by *subjective* assessments?**

☐ yes: participants were asked for subjective description of noise exposure:
    ☐ at home
        ☐ during day
        ☐ at night
    ☐ roads/traffic
    ☐ airplanes
    ☐ other: ___________________
☐ yes: personal feeling of noise annoyance/disturbance (degree with Likert scale etc)
☐ no

- **Details of the noise assessments (complete table with type of assessment, timing, and rough number of assessments):**

**Type of noise assessment**		**Pregnancy (mother)**			**Birth (mother or child)**	**Post natal (child)**	
Noise	Type of measurement	1st trim.	2nd trim.	3rd trim.		Age	Number
Completed:							
Planned:							

B10. POPs

- **Was exposure to POPs (PCBs, dioxins, etc.) assessed in biological samples for the members of your cohort?**

☐ yes
☐ not yet, but planned. Please give predicted year of completion: 20_____
☐ no (proceed to part B11)

- **Which (groups of) POPs were assessed?**

☐ aldrin
☐ chlordane
☐ DDT and metabolites
☐ dieldrin and endrin
☐ heptachlor
☐ hexachlorobenzene
☐ mirex
☐ polychlorinated biphenyls
☐ polychlorinated dibenzo-p-dioxins
☐ polychlorinated dibenzo furans
☐ toxaphene
☐ brominated flame retardants
☐ flourinated compounds
☐ organometallic compunds (TBT)
☐ other: ___________________________________

- **Details of the assessments (complete table with POP type, medium, person, timing, and rough number of assessments):**

**Type of POP assessment**		**Pregnancy (mother)**			**Birth (mother or child)**	**Post natal (child)**	
POPs	Medium	1st trim.	2nd trim.	3rd trim.		Age	Number
Completed:							
Planned:							

- **Was fatty acids measured in the blood samples?**

☐ yes, specify which __________________
☐ no
☐ not yet, but planned

B11. Occupation

- **B11a. Was occupational history collected for the members of your cohort?**

☐ yes
☐ not yet, but planned. Please give predicted year of completion: 20_____
☐ no (proceed to B11.b)

- **Please tick below the periods for which this data was collected and whether it is available for mother and father? Please indicate whether data was collected *prospectively* or *retrospectively*?**

	**Before pregnancy**	**Pregnancy**			Retrospective or prospective	Timing of the questionnaire
		1st trimester	2nd trimester	3rd trimester		
Mother						
Father						

- **Indicate below the codes used for recording occupation and industrial activity in your cohort:**

☐ occupation (ie ILO 1968, or national coding system): __________________
☐ industrial activity (ie ISIC 1971):
☐ no coding, but checklist of occupations
☐ no coding, but recording of job title and/or task performed

- **B11.b Was any specific occupational exposure assessed?**

☐ yes
☐ not yet, but planned. Please give predicted year of completion: 20_____
☐ no

- **If yes, specify the list of occupational exposures assessed** *(ie solvents, hair sprays, mercury, endocrine disruptors, etc…):*

____
____

- **Which type of assessment was used?** (*copy the section for each category of exposure*):

☐ biological sampling
☐ environmental sampling
☐ questionnaires on specific exposures or jobs (e.g. health care workers, hairdressers, agriculture, etc)
☐ Job Exposure Matrix (JEM)
☐ expert judgment
☐ other:__________________

- **Please provide details for the specific occupational exposures. Please indicate whether data was collected prospectively or retrospectively?**

Assessment	**Before pregnancy**	**Pregnancy**			Retrospective or prospective
		1st trimester	2nd trimester	3rd trimester	

B11c. Other data available at a national level:

- **Are you aware of any JEMs built in your country in the recent period (covering the enrolment period of your cohort)?**

☐ yes
☐ no (please proceed to part B12)

- **Which exposures were assessed?**

  ____

- **Which coding system was used?**

  ____

- **Please give a reference or report number describing these JEMs:**

____

____

B12. Other chemical exposures: e.g. BPA, phthalates, etc.

- **Was exposure to any other chemicals assessed for the members of you cohort?**

☐ yes
☐ not yet, but planned. Please give predicted year of completion: 20_____
☐ no

- **Which agents were/will be assessed?**

☐ bisphenol A
☐ phthalates
☐
☐
☐

- **Which type of assessment was used?**

☐ biological sampling
☐ environmental sampling (dust, etc…)
☐ questionnaire data (use of hair sprays, cosmetics, food containers,...), please specify__________
☐ occupational exposure / JEM
☐ dietary exposure
☐ other: _________________

- **Details of the assessments (complete table with type of substance, medium, person, timing, and rough number of assessments):**

**Type of assessment**		**Pregnancy (mother)**			**Birth (mother or child)**	**Post natal (child)**	
Pesticide	Medium	1st trim.	2nd trim.	3rd trim.		Age	Number
Completed:							
Planned:							

C. Health Outcome Assessment

C1. Reproduction and Birth outcomes

- **Was data on reproductive and birth outcomes collected for the members of your cohort?**

☐ yes
☐ not yet, but planned. Please give predicted year of completion: 20____
☐ no (proceed to part C2)

- **Use of a contraceptive method at the start of a pregnancy:**

☐ yes
☐ not yet, but planned. Please give predicted year of completion: 20_____
☐ no

- **Time to pregnancy:**

☐ yes
☐ not yet, but planned. Please give predicted year of completion: 20_____
☐ no

- **Infertility treatment before the index pregnancy:**

☐ yes
☐ not yet, but planned. Please give predicted year of completion: 20_____
☐ no

If yes, was the duration of the pregnancy attempt until the start of the infertility treatment recorded? ☐ yes
☐ no

- **Congenital anomalies:**

☐ yes. Please give %/number of subjects for whom this information was collected: _____
☐ not yet, but planned. Please give predicted year of completion: 20_____
☐ no

- **Specific anomalies of the male reproductive system:**

☐ yes.
  ☐ cryptorchidism (if planned, year _____)
  ☐ hypospadias (if planned, year _____)
  ☐ anogenital distance (if planned, year _____)
☐ no

- **Spontaneous abortions (until 21 weeks of amenorrhea)**

☐ yes. Please give %/number of subjects for whom this information was collected: _____
☐ not yet, but planned. Please give predicted year of completion: 20_____
☐ no

- **Stillbirths (after 22 weeks of amenorrhea)**

☐ yes. Please give %/number of subjects for whom this information was collected: _____
☐ not yet, but planned. Please give predicted year of completion: 20_____
☐ no

- **Medical termination of pregnancy**

☐ yes. Please give %/number of subjects for whom this information was collected: _____
☐ not yet, but planned. Please give predicted year of completion: 20_____
☐ no
If yes, please indicate if information on the reason of the termination of the pregnancy is known, and if the presence of congenital malformations has been recorded.
____

- **Birth weight**

☐ yes. Please give %/number of subjects for whom this information was collected: _____
☐ not yet, but planned. Please give predicted year of completion: 20_____
☐ no
If yes, please indicate how the data were collected:
☐ Self-reported from mothers
☐ Medical record, midwife or doctor reported
☐ Other, specify__________________________

- **Gestational Duration**

☐ yes. Please give %/number of subjects for whom this information was collected: _____
☐ not yet, but planned. Please give predicted year of completion: 20_____
☐ no
If yes, what is the origin for the calculation of gestational duration?
☐ self-reported last menstrual period (by study subject)
☐ Medical record: midwife or physician assessed last menstrual period (on basis of self-report, but assessed and recorded by medically qualified person)
☐ ultrasound
☐ other:

- **Premature Rupture of Membranes**

☐ yes. Please give %/number of subjects for whom this information was collected: _____
☐ not yet, but planned. Please give predicted year of completion: 20_____
☐ no

- **Onset of labour (spontaneous, induced, caesarean section before onset, ...)**

☐ yes. Please give %/number of subjects for whom this information was collected: _____
☐ not yet, but planned. Please give predicted year of completion: 20_____
☐ no

- **Mode of delivery (spontaneous vaginal birth, operative vaginal birth, caesarean section)**

☐ yes. Please give %/number of subjects for whom this information was collected: _____
☐ not yet, but planned. Please give predicted year of completion: 20_____
☐ no

- **Ultrasound measurements**

☐ yes. Please give %/number of subjects for whom this information was collected: _____
☐ not yet, but planned. Please give predicted year of completion: 20_____
☐ no
If yes, describe how many ultrasounds, which gestational weeks:

- **Doppler measurements (of uterine, umbilical, fetal cervical arteries, or other)**

☐ yes. Please give %/number of subjects for whom this information was collected: _____
☐ not yet, but planned. Please give predicted year of completion: 20_____
☐ no
If yes, describe the arteries concerned, which gestational weeks, how many (or percentage of) women:

C2. Neurodevelopment

- **Was data on neurodevelopmental and behavioural outcomes collected for the members of your cohort?**

☐ yes
☐ not yet, but planned. Please give predicted year of completion: 20____
☐ no (proceed to part C3)

- **Which of the following outcomes were assessed in the children:**

☐ neuropsychological assessment (e.g. developmental tests for executive function, memory, language, IQ)
☐ behaviour (ADHD symptoms, etc)
☐ autism symptoms
☐ school achievements/performance
☐ neurophysiology/neuroimaging: _______________
☐ other:_____________________________________

- Details of neurobehavioural and cognitive development assessment of child (number completed or planned)

**Name of test/assessment and year** (Bayley, McCarthy, Griffith, ...)	**Birth**	**Post natal (give months/years of age)**				
		e.g. 14 months		4 years		
**Dubowitz**						
**Bayley** scales of infant development (BSID)						
**Griffiths** Mental Development scales						
**McCarthy** scales of children's abilities (MSCA)						
Wechsler Preschool and Primary scale of Intelligence (**WPPSI**)						
Others:						

- Which of the following assessments were completed in ***mothers and fathers***:

**Type of assessment**	**Name of test**	**Timing**	**Number/% of cohort**
maternal IQ			
paternal IQ			
maternal mental health			
paternal mental health			
maternal stress			
Paternal stress			

maternal attachment			
paternal attachment			
Other			

C3. Allergies and Asthma

- **Was data on asthma and allergies collected for the members of your cohort?**

☐ yes
☐ not yet, but planned. Please give predicted year of completion: 20____
☐ no (proceed to part C4)

- **Which of the following outcomes were assessed in the children:**

☐ asthma
☐ allergies (other than allergic rhinitis)
☐ allergic rhinitis (indoor/outdoor)
☐ eczema
☐ respiratory infections (upper/lower)
☐ food allergies

Asthma

- **Which of the following methods to assess asthma were used?**

☐ parental questionnaires / interview (wheezing, asthma symptoms)
☐ doctor´s diagnosis of asthma (by study doctor or parent-reported doctor´s diagnosis)
☐ lung function tests
  - ☐ oscilometry
  - ☐ spirometry
  - ☐ bronchial challenge test
  - ☐ tested reversibility (bronchodilators)
  - ☐ interrupter technique (Rint)
  - ☐ exhaled NO

Allergic Rhinitis

- **Which of the following methods to assess allergies were used?**

☐ parental questionnaires/ interview (sneezing, runny nose, nasal congestion, itching of the nose, and post nasal drip)
☐ doctor´s diagnosis of allergy (by study doctor or parent-reported doctor´s diagnosis)
☐ sensitization assessment (blood samples, SPT (skin prick test), urine samples)

Eczema

- **Which of the following methods to assess eczema were used?**

☐ parental questionnaires/ interview
☐ doctor´s diagnosis of allergy (by study doctor or parent-reported doctor´s diagnosis)

Allergic Sensitization Assessment

- **Were IgE-antibodies to common inhalant allergens analysed in biological samples?**

☐ yes
☐ no

If yes, describe the specific IgE measured:
  - ☐ total IgE
  - ☐ IgE mite
  - ☐ IgE cat
  - ☐ IgE dog
  - ☐ IgE pollen
  - ☐ IgE grass
  - ☐ other, including food allergies

- **Were skin prick tests (SPT) performed?**

☐ yes
☐ no

If yes, indicate the specific SPT performed:

- ☐ mite
- ☐ cat
- ☐ dog
- ☐ pollen
- ☐ mould
- ☐ others, including food allergens

- **Details of asthma and allergy assessment of child (number completed or planned)**

**Type of assessment and timing**		**Birth**	**Post natal (give months/years of age)**			
Outcome	Method					**Number/% of cohort**
Completed:						
Planned:						

C4. Cancer

- **Is information on childhood cancers collected for your cohort**

☐ yes
☐ not yet, but planned. Please give predicted year of completion: 20_____
☐ no

- **Please describe how:**

☐ linkage to cancer registry
☐ other: ________________________________

- **What is the estimated annual number of childhood cancer cases in your cohort**

0-1 years: _________________
1-2 years: _________________
2-5 years: _________________
5-10 years: _________________
10-15 years: _________________
15-18 years: _________________
(or other, convenient, age categories)

- **Are genotoxicity markers measured in your cohort?**

☐ yes
☐ not yet, but planned. Please give predicted year of completion: 20_____
☐ no

Details?

C5. Childhood growth and obesity, sexual maturation, other outcomes

- **Is information on childhood growth, obesity, sexual maturation, or other metabolic and endocrine disorders, collected for your cohort?**

☐ yes
☐ not yet, but planned. Please give predicted year of completion: 20_____
☐ no, please go to section D

- **Which of the following outcomes were assessed in the children:**

☐ childhood growth and obesity
☐ indicators of metabolic syndrome
☐ diabetes
☐ sexual maturation
☐ other:_____________________________________

- **Details of childhood growth and obesity assessments** (give number/% completed or planned)

**Measure of growth/ body composition**	**Type of assessment (self-report, medical record, measurement, etc)**	**Birth**	**Post natal (give months/years of age)**				
Weight							
Height							
Waist circumference							
Arm circumference							
Wrist circumference							
Fat/fat free mass by bioimpedance							
Other measure of body composition:							

- **Details of metabolic syndrome indicator assessments in children** (give number/% completed or planned)

**Indicator**	**Type of assessment (include whether fasting samples...)**	**Birth**	**Post natal (give months/years of age)**				
Blood pressure							
Cholesterol							
Cholesterol							
Triglycerides							
Glucose							
Insulin							
Other:							

- **Details of sexual maturation assessments** (give number/% completed or planned)

Measure	Type of assessment (self-reported child/mother, evaluated by doctor,...)	Birth	Post natal (give years of age)				
Tanner stage							
Puberal Development Stage							
Age at							
Age at voice change							
Gonadal axis hormones							
Other:							

- **Other outcome assessments, including other biomarkers of effect** (e.g. thyroid hormones, CRP, etc)

Measure	Type of assessment	Prenatal (mother)	At birth	Post natal (give months/years of age)				

D. Other information – including genetic and important covariates - all please comment

D1. Genotyping:

- **Have genetic analyses been performed**

☐ yes, GWAS
☐ yes, specific genes: ____
☐ not yet, but planned. Please give predicted year of completion: 20_____
☐ no

D2. Residential history and time-activity (tick which are available)

- **Home addresses available:**

☐ only once:
 ☐ during pregnancy
 ☐ at birth
 ☐ during follow-up: week/month:_________________
☐ residential history

- **Work addresses of mother during pregnancy:**

☐ yes
☐ no

- **School/daycare addresses of child**

☐ yes
☐ no

- **Were these addresses geocoded?**

☐ yes, specify which _________
☐ not yet, but planned. Please give predicted year of completion: 20_____
☐ no

D3. Time activity patterms

- **Was information on time-activity patterns collected:**

☐ for child
 ☐ questionnaire, specify when _________
 ☐ diary, specify when ____________
☐ for mother
 ☐ questionnaire, specify when _________
 ☐ diary, specify when ____________

D4. Sociodemographic variables

☐ mother's social class (coded from occupation), specify coding system ____________
☐ father's social class (coded from occupation), specify coding system ____________
☐ household income
☐ mother's education
☐ father's education
☐ mother's ethnic origin/country of birth
☐ father's ethnic origin/country of birth
☐ maternal age
☐ paternal age
☐ parity
☐ birth order
☐ child's sex

D5. Breastfeeding

☐ weeks of breastfeeding
☐ weeks of exclusive breastfeeding

D6. Diet and physical exercise

- **Dietary assessments**

☐ yes:
    ☐ FFQ
    ☐ 24 hour recall
    ☐ other: ______________________________
    person (child/mother): ____
    timing (e.g. stage of pregnancy, age of child): _______________________________
☐ no

- **Assessment of physical exercise:**

☐ yes:
    ☐ questionnaire
    ☐ measurements
    person (child/mother): ____
    timing: ____
☐ no

D7. Medical history

- **Is the following information collected for the parents?**

☐ family history
☐ pre-pregnancy medical history of mother
☐ pregnancy complications
        ☐ blood pressure measurements
        ☐ maternal hypertension
        ☐ preeclampsia
☐ maternal allergic history
☐ paternal allergic history

D8. Parental anthropometry

☐ maternal pre-pregnancy weight, height
☐ maternal pregnancy weight, height
☐ paternal weight/height

D9. Other/Comments

Annex 2: Assessment of birth outcomes in European birth cohorts participating in ENRIECO [a]

Table 1. Reproductive and birth outcomes [a] (* indicates that measurements are planned or ongoing but not completed).

Cohort	Time to pregnancy	Congenital anomalies	Spontaneous abortion/stillbirths/terminations	Gestational age	Birth weight	Mode of delivery	Ultrasound measurements
ABCD	X	X	X	X	X	X	
ALSPAC	X	X	X	X	X	X	X
ArcRisk-Norway	X	X		X	X	X	X
BAMSE	X			X	X	X	
BiB		X		X	X	X	X
Co.N.ER	X	X	X	X	X	X	
Czech	X	X	X	X	X	X	
DARC				X	X	X	
DNBC	X	X	X	X	X	X	X
Duisburg		X		X	X	X	
EDEN	X	X	X	X	X	X	X
ELFE	X*	X*	X*	X*	X*	X*	X*
Faroes	X	X	X	X	X	X	X
FLEHS I	X		X	X	X	X	
GASPII				X	X	X	
Generation R	X	X	X	X	X	X	X
Generation XXI	X	X		X	X	X	X
GINIplus				X*	X	X*	
HUMIS	X	X	X	X	X	X	X
INMA old	X	X	X	X	X	X	
INMA new	X	X	X	X	X	X	X
INUENDO	X	X*	X	X	X		
KANC	X	X*		X	X		X
KOALA	X	X		X	X	X	
Krákow		X		X	X	X	
Leicester				X	X	X*	
LISAplus				X	X	X	
LUKAS				X	X	X	
MAS				X	X	X	
MoBa	X	X	X	X	X	X	X
NINFEA	X*	X*		X*	X*	X*	
PARIS	X		X*	X	X	X	
PCB cohort			X	X	X	X	
PELAGIE	X	X	X	X	X	X	
PIAMA				X	X	X	
REPRO_PL		X	X	X	X	X	X
RHEA	X		X	X	X	X	X

[a] Details of each assessment (method, time period, number of subjects) are available on www.birthcohortsenrieco.net (Inventory of ENRIECO Cohorts 2011).

Table 2. Neurodevelopment outcomes [a] (* indicates that measurements are planned or ongoing but not completed).

Cohort	Cognitive function	Behaviour	Autism symptoms	Hyperactivity disorders	School achievements/performance	Mental health	Personality
ABCD	X*	X		X*	X*		
ALSPAC	X	X	X	X	X	X	X
ArcRisk-Norway							
BAMSE							
BiB					X*		
Co.N.ER							
Czech							
DARC							
DNBC		X	X	X	X	X	X
Duisburg	X	X		X			
EDEN	X	X		X			
ELFE	X*						
Faroes	X	X	X	X	X	X	
FLEHS I	X	X	X	X			
GASPII	X	X					
Generation R	X	X	X	X			
Generation XXI							
GINIplus		X		X			
HUMIS	X*	X*	X*	X*			
INMA old	X	X		X			
INMA new	X	X*	X*	X*			
INUENDO				X*			
KANC							
KOALA		X	X	X	X*		
Krákow	X	X	X	X			
Leicester							
LISAplus		X	X	X			
LUKAS							
MAS	X						
MoBa	X*	X*	X*	X*			
NINFEA	X*			X*			
PARIS							
PCB cohort	X	X	X	X			
PELAGIE							
PIAMA					X		
REPRO_PL	X*						
RHEA	X						

[a] Details of each assessment (method, time period, number of subjects) are available on www.birthcohortsenrieco.net (Inventory of ENRIECO Cohorts 2011).

Table 3. Asthma, allergy, and respiratory outcomes, cancer, growth and obesity, metabolic syndrome, sexual maturation [a] (* indicates that measurements are planned or ongoing but not completed).

Cohort	Allergies & Asthma						Cancer	Growth and obesity	Metabolic syndrome	Sexual maturation
	**Asthma**	**Allergies**	**Allergic rhinitis**	**Eczema**	**Respiratory infections**	**Food allergies**				
ABCD	X			X				X	X	
ALSPAC	X	X	X	X		X	X	X	X	X
ArcRisk-Norway										
BAMSE	X	X	X	X	X	X		X		X
BiB								X		
Co.N.ER	X	X	X	X	X	X		X		
Czech	X	X	X		X		X			
DARC	X	X		X	X	X		X		
DNBC	X		X	X	X	X	X	X	X	X
Duisburg	X	X	X	X	X	X		X		X*
EDEN	X	X*	X	X	X	X	X	X	X	
ELFE	X*	X*	X*	X*	X*	X*	X*	X*	X*	X*
Faroes	X		X	X				X	X	X
FLEHS I	X	X	X	X	X			X		
GASPII	X	X	X	X	X	X		X		
Generation R	X	X		X	X	X		X	X	
Generation XXI	X	X	X	X	X	X	X	X	X	
GINIplus	X	X	X	X				X	X	X
HUMIS	X		X	X	X	X	X	X		
INMA old	X	X	X	X	X			X	X	X*
INMA new	X*	X*	X*	X	X	X	X*	X	X	X*
INUENDO								X*		
KANC										
KOALA	X	X	X	X	X	X		X	X	
Krákow	X	X		X	X			X		
Leicester	X	X	X	X	X			X	X*	
LISAplus	X	X	X	X	X	X		X	X	X
LUKAS	X	X	X	X	X	X		X	X	X*
MAS	X	X	X	X		X		X		X
MoBa	X		X	X	X	X	X	X	X	
NINFEA	X*		X*	X*	X*	X*	X*	X*		X*
PARIS	X	X			X			X		
PCB cohort	X	X		X		X		X*	X*	
PELAGIE	X	X	X	X	X	X		X	X	
PIAMA	X	X	X	X	X	X		X	X	X
REPRO_PL	X	X	X	X	X		X	X		
RHEA	X		X	X	X	X	X*	X		

[a] Details of each assessment (method, time period, number of subjects) are available on www.birthcohortsenrieco.net (Inventory of ENRIECO Cohorts 2011).

References

Inventory of ENRIECO Cohorts. 2011. Homepage. Available: http://www.birthcohortsenrieco.net/. [accessed 6 July 2011].
